# Synthesis of Carbon-Zinc Oxide Microspheres Decorated with Ammonium Polyphosphate (APP) for Synergistic Flame Retardancy in Polypropylene Composites

**DOI:** 10.3390/polym17212878

**Published:** 2025-10-29

**Authors:** Juan J. Mendoza, Jesús R. Campos, Ramón Enrique Díaz de León-Gómez, Luciano da Silva, Antonio Serguei Ledezma-Pérez, Arxel de León, Edgar Nazareo Cabrera-Álvarez

**Affiliations:** 1 Centro de Investigación en Química Aplicada (CIQA), Blvd. Enrique Reyna #140, Col. San José de Los Cerritos, Saltillo 25294, Coahuila, Mexicojesus.campos.m24@ciqa.edu.mx (J.R.C.); ramon.diazdeleon@ciqa.edu.mx (R.E.D.d.L.-G.); luciano.dasilva@ciqa.edu.mx (L.d.S.); antonio.ledezma@ciqa.edu.mx (A.S.L.-P.); 2 Secretaría de Ciencia, Humanidades, Tecnología e Innovación (SECIHTI), Av. Insurgentes Sur 1582, Col. Crédito. Constructor, Demarcación Territorial Benito Juárez, Ciudad de Mexico 03940, Mexico; 3 SECIHTI-CIQA, Blvd. Enrique Reyna #140, Col. San José de Los Cerritos, Saltillo 25294, Coahuila, Mexico; 4 SECIHTI-CIQA Monterrey, Alianza Sur 204, PIIT, Apodaca 66628, Nuevo León, Mexico

**Keywords:** polypropylene composites, carbon-zinc oxide microspheres, ammonium polyphosphate, hydrothermal synthesis, flame retardancy

## Abstract

A key strategy for improving polypropylene (PP) fire safety involves developing composites with enhanced flame-retardant properties. In this study, novel flame-retardant systems were developed through the sustainable synthesis of carbon microspheres (CMSs), carbon-zinc oxide microspheres (CZnMSs), and zinc oxide microspheres (ZnMSs). These microspheres were subsequently combined with ammonium polyphosphate (APP) to form synergistic flame-retardant grenades (FRGs). The FRGs were characterized using XRD, FTIR, UV-Vis, TGA, and SEM, and then incorporated into a PP matrix via melt mixing to produce PP-FRG composites. The composites were systematically evaluated for chemical interactions (FTIR), thermal stability and crystallinity (TGA/DSC), morphology (SEM), flammability (UL-94 and cone calorimetry), and mechanical performance (flexural testing). The results demonstrated that the incorporation of FRG in low concentrations (10 wt.%) led to a synergistic effect, improving both fire resistance and mechanical performance of PP-FRG composites compared to neat PP. Among all formulations, the PP-CZnMS/APP composite exhibited the most balanced behavior, combining effective flame inhibition, enhanced char formation, and improved structural integrity.

## 1. Introduction

The constant improvement and technological innovation of polymer-based composite materials have driven the search for formulations that address critical issues, such as the flammability risk of many polymers. In the case of polyolefins, particularly PP, which has wide applications in various industries (electrical, transportation, construction, food, packaging, etc.) [1], the development of materials with flame retardancy is of high concern due to the ease with which this polymer burns, which limits its performance and applicability [2]. In response, flame-retardant systems are commonly integrated into PP composites to mitigate this limitation [3].

In recent years, researchers have explored various flame retardants for PP that not only provide high efficiency in flame retardant processes but also exhibit low smoke generation and reduced toxicity, and offer low cost [4]. Intumescent flame retardants (IFRs), particularly those based on phosphorus, either alone or in combination with other elements, have played a significant role in achieving satisfactory flame retardancy characteristics in PP [5]. IFRs, consisting of an acid source, carbon source, and gas source, function by forming a char layer or barrier on the polymer surface, inhibiting heat transfer and reducing the diffusion of flammable gases [3]. APP is a well-known intumescent flame retardant that is low-cost, highly processable, effective at low concentrations, and environmentally friendly [6]. However, its low compatibility with PP, due to their differing polarities, and its inability to form a stable char limit its performance. Since char formation is essential for APP intumescent action, its effectiveness in PP remains insufficient. To overcome this limitation, APP is often combined with other materials to create synergistic flame-retardant systems, enhancing flame retardancy while improving, or at least not compromising, the mechanical properties of the polymer matrix [5,7,8]. Therefore, developing new IFR systems, including combined flame-retardant components with APP, remains a high-potential area for improvement. The use of APP in combination with metal oxides or carbon-based compounds to form hybrid flame retardants enables the design of innovative strategies to improve the performance of PP, extending its utility in advanced applications [3,5,9,10].

CMSs have emerged as a promising option for combining carbon-based materials with IFRs, due to their ease of preparation and advantageous properties, such as a high specific surface area, adjustable particle size, and chemical stability. The combination of CMSs with IFRs in polymer composites can also improve mechanical properties and thermal stability, and they are considered more environmentally friendly than traditional flame retardants. CMSs are commonly synthesized via pyrolysis, chemical vapor deposition (CVD), or hydrothermal carbonization (HTC). The hydrothermal method stands out as a sustainable, versatile, and cost-effective approach [11]. It transforms organic precursors (e.g., glucose) or biomass into carbon-based spheres with tailored structures (core–shell, hollow spheres, bars, etc.) and properties [12,13]. Jian et al. [14] reported the preparation of composites of polyethylene terephthalate with surface-modified CMSs; the CMSs were synthesized via hydrothermal method using cyclodextrin as the carbon source, followed by surface modification with plasma technology and the grafting of guanidine phosphate (GDP) to form a new IFR. The composites achieved a UL-94 V-0 rating with a 3% load of the intumescent flame retardant and a limiting oxygen index (LOI) value of 32.4%. Qin et al. [15], reported APP-coated CMSs, obtained from glucose by the hydrothermal method, forming a three-in-one IFR for polyester fabric. The results showed that the LOI value of CMS-APP-coated polyester fabric was 28.1%, achieving a B1 class of GB/T 17591-2006, thus broadening the scope of application of this IFR type. Li et al. [16] prepared glucose-derived carbon nanospheres (CNSs) with uniform morphology and good dispersibility via the hydrothermal method and used them to prepare PET composites. The flame-retardant potential of the CNSs was investigated, yielding good performance with an LOI value of 26.3% and a vertical combustion class of V2. Moreover, the introduction of CNSs reduced the heat release rate of PET and promoted the formation of char. The use of CMSs in enhancing the fire safety performance of thermoplastic polyurethane (TPU) composites has also been investigated [17]. Recently, the use of xylose-derived CMSs with different architectures to improve flame retardancy in PP has been reported as a novel synergistic flame-retardant system, making notable progress in this research area [18,19,20].

One of the main reasons CMSs are commonly used in combination with other components, specifically with IFRs, is to boost the flame-retardant effect and avoid the excessive black smoke typically generated when CMSs are incorporated alone into a polymer matrix, which is undesirable for human health and reduces visibility in fire scenarios [21]. A strategy to address this and other critical issues, such as the high load required to enhance the flame-retardant effect significantly, is to combine CMSs with inorganic particles or metal oxides (MO) (e.g., ZnO, TiO_2_) [22]. This CMS-MO synergy leads to the formation of solid or hollow nano- and microstructures, which can enhance flame retardancy during the combustion process of polymers. One of the most common methods for synthesizing these CMS-MO structures involves creating templates that may or may not be removed by post-treatment or by sacrificing the core [22]. These modified CMSs with metal oxides are highly applicable in various fields, including energy storage (e.g., supercapacitors), catalysis, sensors, and environmental remediation [23,24,25,26]. In terms of flame-retardant function, zinc oxide (ZnO) has garnered considerable interest as a suitable synergistic agent, not only due to its smoke suppression effects but also for its potential to catalyze char formation, non-toxicity, and low production cost [27]. The use of ZnO as a synergist with IFRs has been explored in various polymeric matrices, such as phenolic foams [28], cotton [29], flexible poly(vinyl chloride) [30], ethylene-vinyl acetate [31], poly(lactic acid) [32], epoxy resins [33,34], thermoplastic polyurethane [35] and PP [36,37,38,39,40,41].

Hu et al. [36] reported the use of ZnO combined with melamine phosphate and incorporated with pentaerythritol to create an intumescent flame retardant used in PP. With the incorporation of ZnO, the LOI value was 10% higher than that of the composites without ZnO. Another IFR, formed by an organic-inorganic hybrid char-forming agent of ZnO modified combined with APP, was reported by Xu et al. [37] to show high efficiency in enhancing flame retardant and smoke suppression properties in PP. The use of ZnO nanowires synthesized via the hydrothermal method and incorporated into PP to prepare ZnO nanowires/PP nanocomposites improved thermal stability while significantly reducing the peak heat release rate (PHRR), total heat release (THR), and maximum smoke density (MSD) of the nanocomposites [38]. Recently, Cheng et al. [39] reported the synergistic effect of ZnO with piperazine pyrophosphate (PPAP)/melamine polyphosphate (MPP) for flame retardancy in PP composites prepared by a melt blending method. The authors observed that ZnO significantly inhibited smoke formation during the combustion process, thereby improving thermal stability and enhancing the strength of the intumescent char layer, which in turn increased the flame retardancy of PP. Additionally, Zhang et al. [40,41], modified zinc oxide (mZnO) with polysiloxanes and investigated its effectiveness as an intumescent flame retardant for PP. By introducing 16 wt.% of the IFR and 0.3 wt.% maleic anhydride grafted PP (MAH-g-PP), the limiting oxygen index increased to 32.7%, and a UL-94 V-0 rating was achieved. Moreover, the material exhibited improved mechanical properties compared to pure PP. The ZnO microencapsulated by polysiloxane contributed to a synergistic enhancement in flame retardancy, mechanical properties, and UV and water resistance in the composites, in addition to the use of compatibilizer, which improves mechanical properties and flame retardancy, as has been reported in other systems [42].

Song Y. et al. [43], synthesized carbon microspheres (CMSs) via a hydrothermal process and were subsequently modified with 3-aminopropyltriethoxysilane (APTS) and dispersed in ammonium polyphosphate (APP). Methacryloxypropyltrimethoxysilane (γ-MPS) was employed to enhance the compatibility of the modified CMS within a polyethylene terephthalate (PET) matrix. The modified CMSs, referred to as CA, exhibited significant improvements in the mechanical properties of the PET matrix. Additionally, a substantial reduction in peak heat release rate (PHRR) and total smoke production (TSP) was observed 71.4% and 18.8%, respectively when the CMS:APP ratio was 1:2. Regarding fire performance, this formulation achieved a V-0 rating in the UL-94 vertical burning test and showed an increase in the limiting oxygen index (LOI) to 28.6%. Zang B. et al. [44] developed ZnO in a traditional intumescent flame retardant system composed of APP, Pentaerythritol, and melamine for styrene-butadiene-styrene. The results demonstrated that ZnO catalyzes the decomposition of APP, promoting crosslinking and enhancing the viscosity of the resulting char layer. Furthermore, it was found that when melamine is included and ZnO is present at low concentrations (0.5%), the effect achieved is the stabilization of the char layer. However, higher ZnO contents (>1%) lead to its rupture or hinder its expansion.

Considering this background, the development of new flame-retardant systems to further improve IFR efficiency and enhance the performance of PP remains a significant research opportunity. Additional efforts are required to design flame retardants with superior synergistic effects. Therefore, this work examines the synthesis of ZnO, CMS, and APP-decorated hollow particles with controlled morphologies using green methodologies, as well as their effect on the flame retardancy properties of polypropylene. In this work, we introduce a novel approach in which dextrose is used as a template to fabricate microspheres via a hydrothermal method, aiming to achieve a synergistic effect with APP. The resulting particles, termed FRG, were synthesized and systematically characterized by XRD, FTIR, UV-VIS, SEM, and TGA. The synthesis pathway involved the preparation of CMS, CZnMS and ZnMS. Finally, the potential of these FRG (10 wt.%) to enhance the thermal stability, mechanical performance, and flame-retardant properties of PP composites was evaluated.

## 2. Materials and Methods

### 2.1. Materials

D-(+)-Dextrose, sulfuric acid (95–98%), zinc acetate (99.6%), ethylene glycol (99.8%), ethanol (99.9%), and other common solvents were acquired from Merck-Aldrich (Darmstadt, Germany). Ammonium polyphosphate (APP *n* ≥ 1000) (Preniphor™ EPFR-APP222H (Qingyuan, China)) was supplied by Presafer. PP (Formolene^®^ 4100N, MFI ≈ 12.9 g/10 min) was sourced from Formosa Plastics (Livingston, NJ, USA).

### 2.2. Methods

#### 2.2.1. Synthesis of CMS

The CMSs were synthesized through a hydrothermal method [45]. Briefly, 0.083 mol of anhydrous dextrose was dissolved in a mixed solution of water/ethylene glycol in a 70:30 *v*/*v* ratio. Then, 0.036 mol of sulfuric acid was added and stirred continuously for 15 min. The homogeneous solution was transferred to a Teflon-lined stainless-steel autoclave, and the reaction was conducted at 180 °C for 4 h. The product was recovered by centrifugation (13,000 rpm, 15 °C, 20 min), washed several times with distilled-deionized water (DDI) and ethanol, and finally freeze-dried. The yield of the CMSs was approximately 42%.

#### 2.2.2. Synthesis of CZnMS and ZnMS

Using the obtained CMSs as a template, ZnO microspheres were synthesized as follows: 3 g of CMS, 12 g of zinc acetate dihydrate (Zn(CH_3_CO_2_)_2_·2H_2_O), and 150 mL of DDI water were sonicated for 10 min. The mixture was placed in a Teflon-lined stainless-steel autoclave and heated at 120 °C for 4 h. After cooling in an ice bath for 30 min, the product was centrifuged at 13,000 rpm for 20 min, washed three times with water and ethanol, and dried in an oven at 60 °C. The product at this stage is the CZnMS. To remove the carbon component, the dried solid underwent a calcination process at 500 °C for 4 h with a heating rate of 1 °C/min. The resulting ZnMSs were obtained with a yield of 5.6%.

#### 2.2.3. Preparation of FRG

Initially, an aqueous suspension of 2% m/v commercial APP was subjected to particle size reduction using an ultrasound probe (33.7 μm amplitude, 60%), followed by filtration to obtain an average particle size of ~11 μm, as shown in the Supplementary Material. The filtered particles were then dried by lyophilization. To prepare the FRG, either CMS, CZnMS or ZnMS were mixed with APP at a 1:5 weight ratio. Solution A was prepared by dispersing 2 g of the required microspheres in 880 mL of ethanol using an ultrasound bath for 30 min, followed by stirring for 24 h. On the other hand, solution B, containing 10 g of APP and 2000 mL of DDI water, was prepared and stirred for 24 h. The FRG was obtained by dropwise addition of solution A to solution B by stirring for 24 h. The resulting FRG was recovered by evaporating the water, drying at 60 °C, washing with water, and finally freeze-drying to obtain a gray solid.

#### 2.2.4. Preparation of PP-FRG Composites

Composites of PP and FRG were prepared by melt mixing. The PP and FRG were mixed in a Brabender ATR Plasti-Corder internal mixer equipped with roller rotors, operating at 60 rpm and 190 °C for 15 min. The resulting blends were pelletized and compression-molded using a hydraulic press at 190 °C under 25 tons of pressure for 5 min to produce square plaques (~3 mm thick, 225 cm^2^ area). The formulations are presented in Table 1.

#### 2.2.5. Characterization and Evaluations

UV-VIS absorbance spectra w|as recorded using a Shimadzu UV-2401PC spectrophotometer (Shimadzu corporation, Kyoto, Japan) with a 200–800 nm wavelength range. Samples were dispersed in ethanol using an ultrasound bath for 5 min before measurements. Fourier Transform Infrared Spectroscopy (FTIR-ATR) spectra were acquired using a Thermo Scientific Nicolet iS10 spectrophotometer (Thermo Fisher Scientific, Waltham, MA, USA), with 32 scans in the 625–4000 cm^−1^ range and a resolution of 4 cm^−1^. X-ray diffraction (XRD) analysis was carried out using a SIEMENS D500 diffractometer (SIEMENS AG, Karlsruhe, Germany), operating at 35 kV and 25 mA (Cu Kα X-ray source, λ = 1.54 Å) with a scan range of 10 to 80° 2θ and a step size of 0.07°. Thermogravimetric analysis (TGA) was conducted using a TGA Q500 V6.7 (TA Instruments, New Castle, DE, USA) Build 203 analyzer. Samples were analyzed from 30–600 °C, followed by a switch to oxygen from 600–700 °C, with a heating rate of 10 °C/min. Scanning Electron Microscopy (SEM) was performed using a JCM-6000 microscope (JEOL Ltd., Akishima, Tokyo, Japan), while Field Emission Scanning Electron Microscopy (FESEM) was conducted on a JEOL JSM-7401F microscope ((JEOL Ltd., Akishima, Tokyo, Japan). LEI (secondary electrons) and COMPO (backscattered electrons) modes were used. Optical microscopy (OM) was performed using a Digital Microscope VHX-5000 (Keyence, Osaka, Japan). Mechanical properties were evaluated according to ASTM D790 [46]. Standard test specimens were obtained from the compressed composite samples, which were conditioned at 23 ± 2 °C and 50% relative humidity for 48 h before testing. A United universal testing machine (Instron 4301, Norwood, MA, USA) with a 500 N load cell was used, and five specimens of each PP-FRG composite formulation were tested, with the average values reported. Flammability tests were conducted in accordance with the UL-94 standard, using specimens with dimensions of 125 × 13 × 3 mm that were previously conditioned at 23 ± 2 °C and 50% relative humidity. Following the ASTM E1354 [47] method, cone calorimetric tests (Fire Testing Technology, East Grinstead, UK) were performed using specimens with dimensions of 100 × 100 × 3 mm; each experiment was conducted under a heat flux of 35 kW/m^2^.

## 3. Results and Discussion

**Figure 1:**
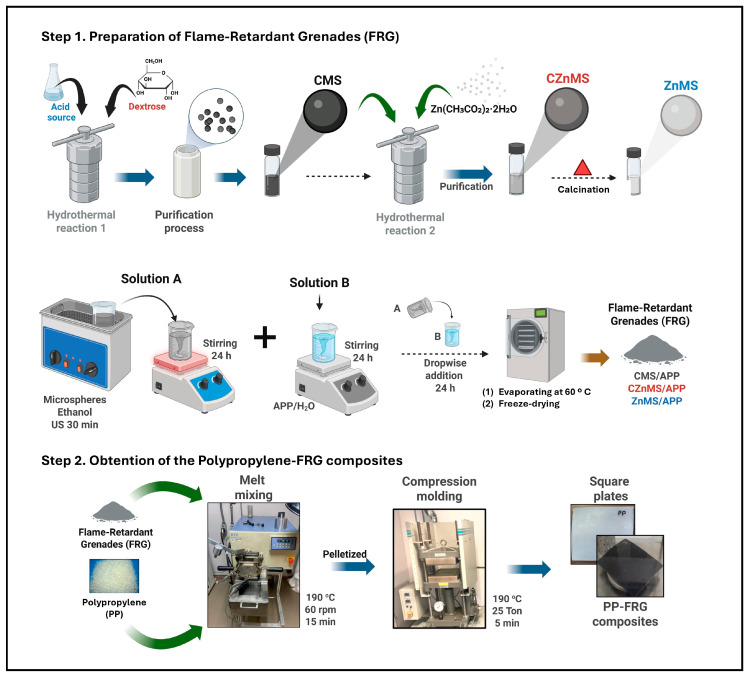
Schematic representation of the two-step synthesis of FRG and the fabrication of PP-FRG composites. CMS, CZnMS, and ZnMS were synthesized via hydrothermal and calcination processes, then decorated with APP to obtain FRG. These FRG were then mixed with PP and compression-molded to produce PP-FRG composite plates.

A general schematic preparation of different FRG and their incorporation into a PP matrix is shown in Figure 1. The preparation involved two main steps. The first step was to synthesize microspheres and subsequently decorate them with APP to achieve a synergistic flame-retardant effect. In the second step, these FRG systems were incorporated into the polymer matrix to obtain the corresponding composites.

CMSs were synthesized via a hydrothermal method, a process that is sustainable, versatile, and cost-effective. This method is based on acid-catalyzed hydrothermal carbonization (HTC) of dextrose, used as the organic precursor. During this process, dehydration reactions generate small molecules such as 5-hydroxymethylfurfural (5-HMF) and other furan derivatives [11,25]. These intermediates then undergo condensation polymerization reactions. The resulting polymer chains (polyfurans and other polymer-rich carbonaceous species) reach supersaturation and agglomerate, forming thermodynamically stable nuclei. These nuclei reduce the surface energy of the system and grow into CMSs with a spherical structure composed of amorphous carbon and oxygen-rich functional groups [12,45].

Using the obtained CMSs as a core or template, zinc oxide was incorporated as a shell by a hydrothermal method, forming CZnMS. The synthesis is based on the formation of Zn^2+^ ions from zinc acetate dihydrate and its coordination via electrostatic interactions between these zinc ions and the oxygen-rich functional groups (e.g., O–H,–COOH) on the surface of the CMS. Under these hydrothermal conditions, the zinc ions hydrolyze and are subsequently oxidized by the temperature and pressure conditions within the reactor, ultimately forming CZnMS, which adopts the spherical morphology of the CMS. Finally, the carbon component is removed under a calcination process, thus yielding the ZnMS [22,26,45].

Next, each of the three microsphere systems (CMS, CZnMS, and ZnMS) was combined with APP to produce the FRG. Finally, these new potential FRG systems were incorporated into the polymer matrix to PP-FRG composites.

### 3.1. Physicochemical Characterization of the Different Microspheres: CMS, CZnMS, and ZnMS

**Figure 2:**
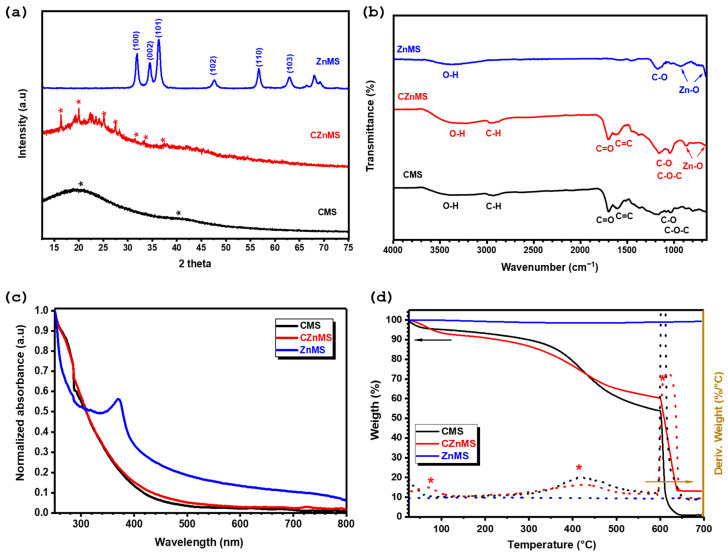
Physicochemical characterization of CMS, CZnMS, and ZnMS: (**a**) X-ray diffraction patterns, (**b**) FTIR spectra, (**c**) UV-Vis absorption spectra, and (**d**) TGA and DTGA analyses. * indicates peaks of interest.

Figure 2 shows the X-ray diffraction (XRD) patterns of the different synthesized microspheres. The XRD pattern of CMSs exhibits two broad halos centered around 21° and 41° in 2θ, with no sharp diffraction peaks detected. This pattern corresponds to the amorphous nature of the CMS, which is typical of microspheres derived from organic precursors through hydrothermal processes [45,48]. In the case of the CZnMS, the XRD pattern also exhibits two broad halos, indicating the predominance of an amorphous structure. However, the appearance of some weak and poorly resolved diffraction peaks suggests a partial transition from an amorphous to a semicrystalline structure, reflecting an increase in the long-range structural order. These present but not well-defined crystalline peaks are likely associated with an intermediate phase formed during the stages of zinc oxide formation, where zinc ions begin to coordinate and oxidize on carbonaceous surfaces before thermal treatment [26,45]. After the calcination process, the XRD pattern reveals a well-defined peaks at 2θ ≈ 31.7°, 34.4°, 36.2°, 47.5°, 56.6°, and 62.8°, corresponding to the (100), (002), (101), (102), (110) and (103) planes of crystalline zinc oxide (JCPDS 36-1451) [45,49]. The complete disappearance of the amorphous carbon signal confirms the successful thermal decomposition and removal of the CMS, resulting in the formation of the ZnMS.

The chemical composition was analyzed by FTIR (Figure 2b). The FTIR spectrum of CMS indicates the presence of various functional groups typically found in carbonaceous materials [16,25]. These include broad O–H stretching vibrations in the range of 3600–3100 cm^−1^ and aliphatic C–H stretching bands at approximately 2959 cm^−1^ and 2880 cm^−1^. The CMS structure with oxygen-rich functional groups exhibits intense bands at 1697 cm^−1^ and 1593 cm^−1^, corresponding to the carbonyl group (C=O) from the carboxylic acid functionality and the C=C bond from the aromatic structures present. Additionally, bands corresponding to C–O and C–O–C stretching are observed around 1160 cm^−1^ and 1030 cm^−1^, respectively. In the same Figure 2b, the FTIR spectra of CZnMS, shows similar absorption bands observed for CMS which implies that the CMS remain mostly intact at this stage, but with some changes, such as the shift in the C=O band to a higher wavenumber at 1702 cm^−1^, indicating association of coordination of this group with zinc ions now present. In addition, the presence of weak absorption bands between 800–600 cm^−1^, confirms the initial Zn–O bond formation as consequence of deposition of zinc oxide on the CMS. After the calcination process, the FTIR spectrum for ZnMS exhibits a significant reduction in the intensity of the absorption bands associated with organic groups from the CMS, such as C–H and C=O, confirming the effective thermal decomposition of the organic template and the formation of ZnMS in the system. The residual O–H and C–O bands suggest the presence of adsorbed water molecules and/or hydroxyl groups on the microspheres surface, likely originating from post-calcination surface rehydration rather than residual CMS. Finally, Zn–O vibrations appear in the low-frequency region between 800–600 cm^−1^, confirming the formation of zinc oxide [50,51]. This ZnMS FTIR spectrum is consistent with the wurtzite structure observed in X-ray diffraction.

UV-Vis spectroscopy was employed to complement the analysis of the chemical composition of the different synthesized microspheres by evaluating the optical and electronic transitions associated with their structures. The normalized UV-Vis spectra for CMS, CZnMS, and ZnMS are shown in Figure 2c. A similar pattern can be observed for CMS and CZnMS, both displaying high absorbance between 250–350 nm that decreases toward higher wavelengths, with a shoulder centered at ~279 nm, attributed to π–π* electronic transitions from the predominant carbonaceous systems, as a result of the presence of aromatic or conjugated structures in both CMS and CZnMS. However, slight differences in the curve profiles and slopes suggest the influence of zinc species in the CZnMS sample. In contrast, the UV-Vis spectrum of ZnMS shows the absence of the shoulder associated with carbon-based materials and instead presents a sharp peak at 370 nm, which corresponds to the band-edge absorption, confirming the formation of ZnMS [24,45].

The synthesized CMS, CZnMS, and ZnMS were also evaluated by thermogravimetric analysis (TGA), which revealed the thermal behavior of the materials. The TGA and derivative thermogravimetric analysis (DTGA) curves are shown in Figure 2d. Below 120 °C, CMS and CZnMS exhibit weight losses of approximately 5% and 7%, respectively, corresponding to moisture adsorbed within the microspheres. The curves remain relatively stable until ~230 °C, after which they begin to decay, showing a change in slope between 230–580 °C in both samples. This behavior is attributed to the decomposition of the organic matter forming the microspheres, generating CO and CO_2_ in the process. The maximum decomposition rates, indicated by the DTGA curves, occur at 418 °C for CMS and 423 °C for CZnMS, suggesting improved thermal stability in the presence of zinc oxide deposited onto the CMS. This enhanced stability is also reflected in the residual mass observed once the analysis atmosphere switches to oxygen, with CZnMS retaining a higher residue (~13 wt.%) compared to the carbon-rich CMS (less than 1 wt.%). Finally, the TGA curve for ZnMS remains unchanged throughout the analysis, indicating no mass loss and confirming the absence of the CMS template and the presence of thermally stable ZnO, as previously verified by XRD, FTIR, and UV-Vis analyses. The DTGA curve also shows no detectable decomposition phase, further supporting the thermal stability of the ZnMS sample.

#### Morphology, Size Dispersion, and EDX Analysis

**Figure 3:**
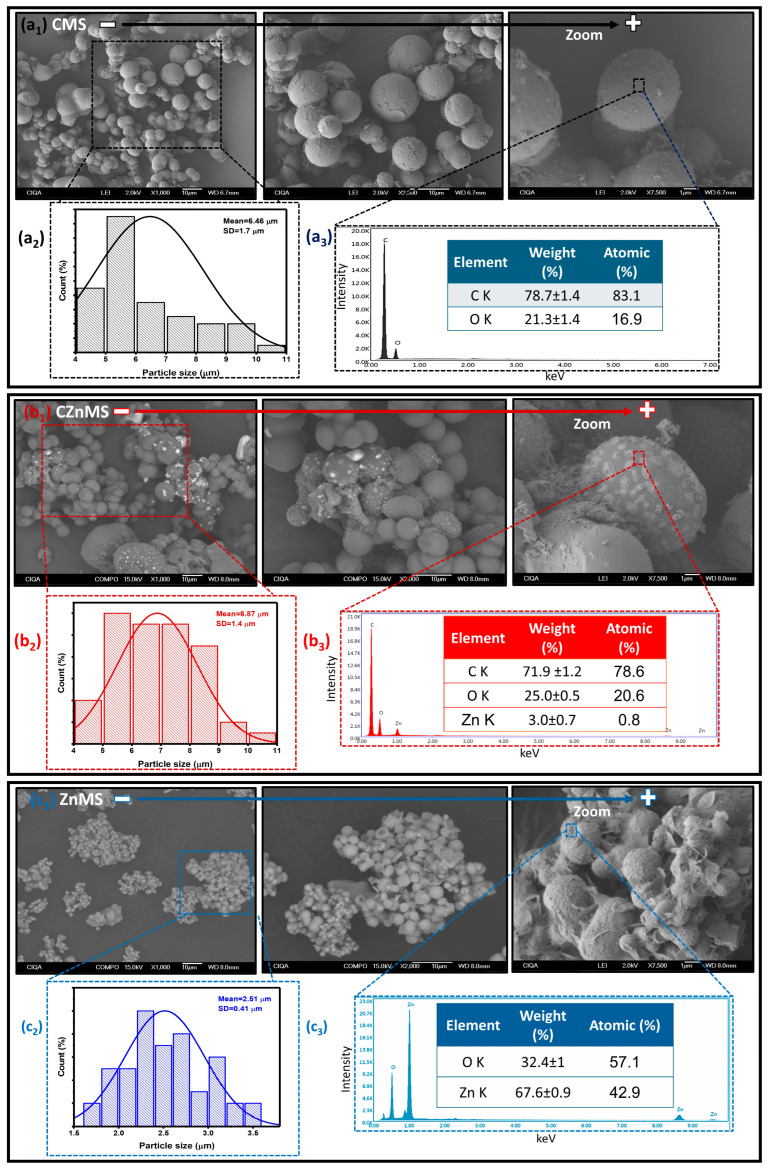
Morphological characterization, particle size distribution, and elemental composition of the synthesized microspheres: (**a_1_**,**b_1_**,**c_1_**) SEM micrographs at 1000×, 2000× and 7500× magnifications; (**a_2_**,**b_2_**,**c_2_**) particle size distribution histograms; and (**a_3_**,**b_3_**,**c_3_**) energy-dispersive X-ray spectroscopy results for CMS, CZnMS, and ZnMS, respectively.

Figure 3 shows SEM micrographs at different magnifications (1000×, 2000×, and 7500×), along with the elemental composition of the different microspheres. The CMS exhibits a uniform spherical morphology with a smooth surface and an average particle size of 6.46 ± 1.7 µm. The carbon-rich composition (78.7 wt.%) was confirmed by energy-dispersive X-ray spectroscopy (EDX). Such morphology is typically reported for carbonaceous microspheres synthesized via hydrothermal carbonization (HTC) of glucose-derived precursors [1,2]. Jian Peng et al. [3], studied the influence of acid and temperature regulation during the HTC process and reported comparable spherical morphologies under similar conditions (160–180 °C, pH ≈ 2). However, in their case, longer reaction times (approximately 8 h) were required to achieve particle sizes comparable to those obtained in this work. The SEM analysis of CZnMS revealed a spherical shape similar to CMS, but with a partially coated and roughened surface, suggesting successful deposition of zinc species onto the carbonaceous structure. This deposition was preserved at this stage, as confirmed by FTIR and XRD analyses. The CZnMS exhibited an average particle size of 6.87 ± 1.4 µm, and the EDX analysis showed the presence of zinc (3 wt.%), confirming the formation of a hybrid carbon/zinc composition. After calcination, the average particle size of the ZnMS decreased significantly to 2.51 ± 0.4 µm, based on statistical analysis of more than fifty particles. This size reduction correlates with the removal of the CMS carbonaceous template and is supported by EDX results, which indicate a composition of 67.6 wt.% zinc and 32.4 wt.% oxygen. The ZnMS exhibit a rougher, slightly porous morphology and are composed entirely of thermally stable inorganic microspheres. These features are consistent with the thermal resistance observed in the TGA analysis.

### 3.2. Flame-Retardant Grenades (FRG)

#### 3.2.1. Characterization by FTIR and TGA

**Figure 4:**
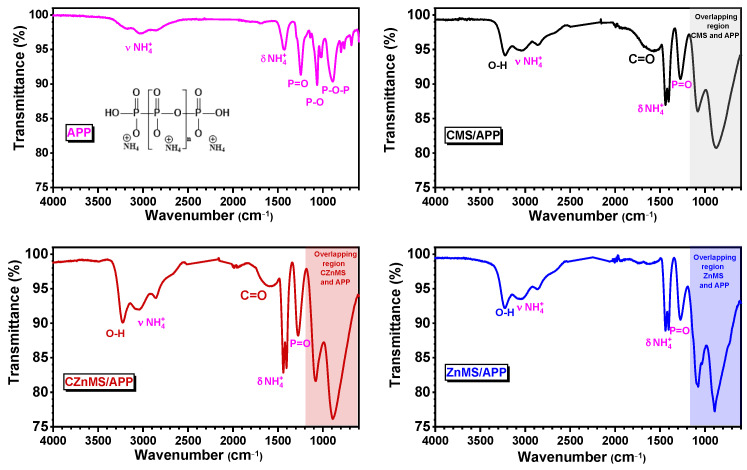
FTIR spectra of APP and the different FRG: CMS/APP, CZnMS/APP, and ZnMS/APP.

The preparation of FRG involved the decoration of previously synthesized microspheres with APP. The FTIR spectra of APP and all FRG formulations are shown in Figure 4. The spectrum of APP exhibits its characteristic absorption bands corresponding to polyphosphate chains and ammonium groups, including the N–H stretching (νN–H) at 3030 cm^−1^, N–H bending (δN–H) at 1430 cm^−1^, and phosphate-related vibrations such as νP=O at 1245 cm^−1^, νP–O at 1064 cm^−1^, and νP–O–P at 890 cm^−1^ [21]. In the case of the FRG labeled as CMS/APP, the FTIR spectrum highlights two main features. First, the primary APP-related bands, particularly those associated with NH_4_^+^, P=O, and P –O–P groups, are preserved with only minor shifts. Second, new signals attributable to the CMS component emerge, including the O–H stretching vibration that overlaps with the N–H band, and a shoulder centered at ~1640 cm^−1^, which is associated with oxygen-containing functional groups (e.g., C=O) on the CMS surface. Additionally, a broad overlapping region below 1100 cm^−1^ highlighted in the shaded gray area suggests physical interactions between the CMS and APP. These interactions are likely mediated by hydrogen bonding, and, since no new bands are observed, non-covalent bonds are inferred.

The FTIR spectrum of CZnMS/APP FRG exhibits a similar behavior to the one mentioned for CMS/APP, with phosphate-related bands of APP and the carbon-oxygen signals of CMS. However, some differences appear: the phosphate bands appear broader, which may originate from the interaction between surface hydroxyl groups of microspheres and the amino functionalities of APP, as well as from the coordination with zinc species. The broadening of phosphate bands, particularly in the red-shaded region below 1100 cm^−1^, suggests stronger interactions, potentially involving both hydrogen bonding and electrostatic interactions between phosphate groups and exposed Zn^2+^ sites. These features imply a hybrid interaction mechanism, where the presence of Zn increases its affinity with APP, resulting in an overlap of signals, as reported in other similar systems [36,37].

In contrast, the FTIR spectrum of ZnMS/APP no longer displays bands associated with the carbonaceous material, as anticipated. The characteristic signals of APP persist, but some modifications are observed in the blue-shaded region, suggesting the presence of chemical interactions, since there is no longer the presence of carbonyl groups of the CMS, it is possible to appreciate the signals at 1075 cm^−1^ of P–O bond and a new over-lapping signal appears at 1025 cm^−1^ due to the Zn–O bond of the ZnO present in the particles, as observed in other systems [31,34]. As previously mentioned, these interactions are likely dominated by the coordination capacity of zinc ions, which facilitates strong interactions between the zinc oxide surface of the microspheres and the phosphate groups that constitute the APP structure, particularly through the formation of Zn–O–P bonds. Overall, these results reveal a progressive increase in interaction strength within the newly developed flame-retardant grenades, from physical adsorption in CMS/APP, to combined physical and covalent interactions in CZnMS/APP, and finally to strong chemical coordination in ZnMS/APP.

**Figure 5:**
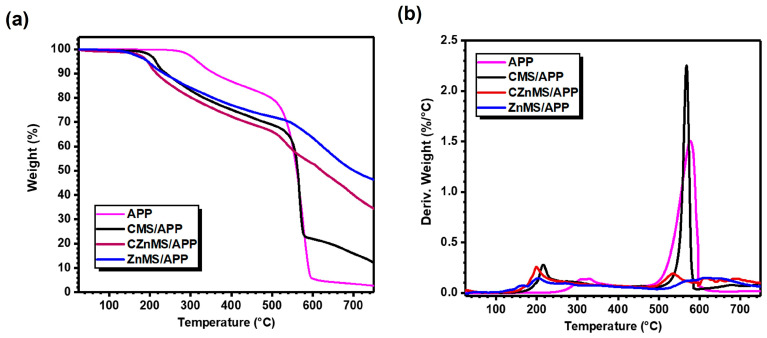
(**a**) TGA and (**b**) DTGA of APP and the different FRG: CMS/APP, CZnMS/APP and ZnMS/APP. The curves highlight differences in thermal stability, degradation steps, and final char yields among the formulations.

Figure 5a shows the thermogravimetric analysis, while Figure 5b presents the derivative thermogravimetric analysis of the obtained FRG, along with that of APP. The latter exhibits stable thermal behavior up to 270 °C, after which decomposition begins. This decomposition occurs gradually in two steps between 270–600 °C, with a sharp onset around 320 °C and a major weight loss step at 576 °C, as observed in the DTGA curve. The first degradation step is attributed to the release of volatile products such as NH_3_ or H_2_O, leading to dehydration and crosslinking of polyphosphates (e.g., polyphosphoric acid, pyrophosphoric acid, and P_2_O_5_) [7,52]. In the second step, the main degradation of APP and decomposition of its derived products takes place, resulting in a total weight loss of approximately 95 wt.% at 600 °C. A final residue of ~2 wt.% remains once the oxidative atmosphere is introduced, completing the thermal decomposition process of APP [15].

In the case of CMS/APP, a similar pattern is observed in both TGA and DTGA curves compared to APP alone, with maximum decomposition rates at 216 °C and 567 °C. However, the inclusion of CMS promotes the char formation due to interactions between phosphorus species from APP and carbonaceous components of the CMS, contributing to a thermal barrier effect. This effect is reflected in a higher residual of 22 wt.% at 600 °C, and a chart residual of ~11 wt.%. For CZnMS/APP and ZnMS/APP, the TGA and DTGA curves reveal a clear synergistic effect, evidenced by slower degradation rates and the formation of a more protective barrier layer during decomposition. This results in the highest residual masses among the systems tested. These features are highly desirable for enhancing flame-retardant performance when these systems are incorporated into polymer matrices.

The improved thermal barrier effect in CZnMS/APP and ZnMS/APP correlates with the strong chemical interaction, particularly electrostatic forces and the Zn–O–P coordination observed in the FTIR analysis. These interactions stabilize the phosphate structure, preserve the integrity of the FRG during heating, with and promote substantial chart formation, reaching 34.3 wt.% and 46.2 wt.% for CZnMS/APP and ZnMS/APP, respectively.

#### 3.2.2. Morphology, EDX Analysis and Elemental Mapping

**Figure 6:**
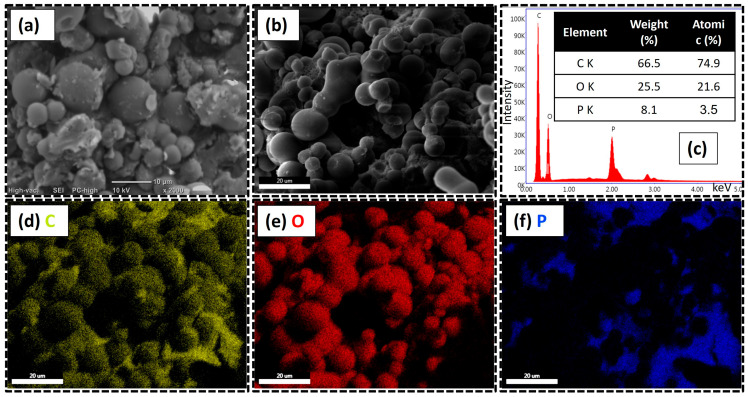
Morphological and elemental characterization of the CMS/APP FRG: (**a**) SEM micrograph, (**b**) FESEM micrograph, and (**c**) EDX spectrum; (**d**–**f**) elemental mapping of carbon (C), oxygen (O) and phosphorus (P), respectively.

The morphology of the CMS/APP FRG was characterized by SEM (Figure 6a) and FESEM (Figure 6b), while elemental analysis was carried out using EDX (Figure 6c). The SEM micrograph shows that the CMS/APP FRG maintains a smooth surface and well-defined spherical shape of the CMS but is now accompanied by some irregular plate-like structures attributed to APP, which also appear relatively smooth. The polyphosphate particles seem to be coated and adhered to the CMS surface, indicating good physical interaction between both components [53,54]. These interactions may be enhanced by the increased surface area of APP, achieved by reducing its average particle size from 20–30 µm to 2–6 µm before FRG formulation, as reported in the Supplementary Material. In contrast, it is reported that the use of unmodified commercial APP often leads to particle aggregation and poor dispersion [7]. The FESEM micrograph confirms the relatively smooth and uniform spherical structure of the CMS/APP FRG, as well as surface adsorption between the two components. The EDX spectrum revealed that the system consists mainly of carbon (66.5 wt.%), oxygen (25.5 wt.%) and phosphorus (8.1 wt.%), reflecting the carbonous nature of CMC and the oxygen-rich content of both CMS and APP. To further confirm this composition, elemental mapping (Figure 6d–f) shows that carbon (C) and oxygen (O) are homogeneously distributed, while phosphorus (P) appears less uniformly dispersed, supporting the assumption that APP is localized on the surface of the CMS or partially agglomerated.

**Figure 7:**
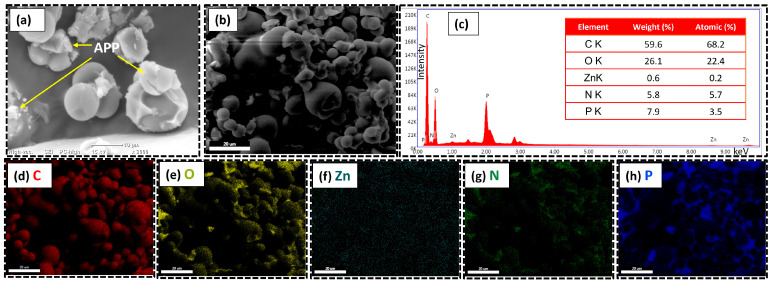
Morphological and elemental characterization of the CZnMS/APP FRG: (**a**) SEM image, (**b**) FESEM image, and (**c**) EDX spectrum; (**d**–**h**) elemental mapping of carbon (C), oxygen (O), zinc (Zn), nitrogen (N), and phosphorus (P), respectively.

The morphology and compositional features of the CZnMS/APP FRG are shown in Figure 7. The SEM image (Figure 7a) reveals the spherical morphology of the microspheres coated with irregular flake-like APP structures. This uniform APP coverage indicates successful integration of both components and suggests strong interfacial interactions. The high-resolution FESEM image (Figure 7b) shows that the particles are interconnected through the presence of APP, revealing a rougher surface compared to the CMS/APP FRG. This improved coating is attributed to enhanced interactions and binding sites between components, likely promoted by the incorporation of zinc domains. The elemental composition was confirmed by EDX analysis (Figure 7c), showing that carbon (59.6 wt.%) and oxygen (26.1 wt.%) remain the dominant elements. Additionally, the presence of zinc (0.6 wt.%), phosphorus (7.9 wt.%), and nitrogen (5.8 wt.%) supports the effective incorporation of APP, forming the new CZnMS/APP FRG structure. The elemental mapping (Figure 7d–h) shows carbon (C), oxygen (O) and zinc (Zn) homogenously distributed, forming the structural backbone of the FRG. Meanwhile, nitrogen and phosphorus are also well dispersed, confirming that the APP is successfully deposited onto the microspheres through a combination of physical and chemical interactions. These findings are consistent with FTIR and TGA results.

**Figure 8:**
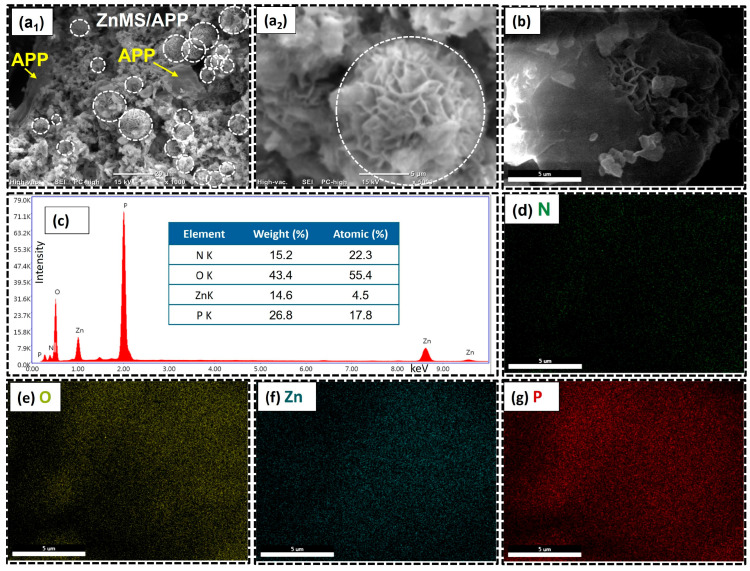
Morphological and elemental characterization of the ZnMS/APP FRG: (**a_1_**,**a_2_**) SEM micrographs at 1000× and 5000× magnifications, respectively; (**b**) FESEM micrograph; (**c**) EDX spectrum; (**d**–**g**) elemental mapping of nitrogen (N), oxygen (O), zinc (Zn), and phosphorus (P), respectively.

The SEM (1000× and 5000×), FESEM micrograph (5000×), elemental mapping, and EDX spectrum for ZnM/APP FRG are shown in Figure 8. As observed in the micrographs, the morphology of this new flame-retardant grenade is quite different from that of the formulations with CMS and CZnMS. The rough surface of the original ZnMS is still present; however, the incorporation of APP results in irregular aggregates distributed over the ZnMS core, decorating the inorganic microspheres and forming numerous surface flakes. This type of morphology has been observed in other systems, such as CMS combined with layered double hydroxide (LDHs) coated with hydrotalcite, containing many flake structures and forming a core–shell morphology [19,20].

To the best of our knowledge, this is the first report of such morphology in a ZnMS-APP system. This behavior is attributed to interfacial interactions involving both physical attachment and chemical bonding. The result is a multilayered surface decorated with APP deposited onto the ZnMS, likely favored by the APP ultrasound treatment and reduction in particle size before FRG formulation. The chemical composition was confirmed by EDX analysis (Figure 8c), showing the presence of zinc (14.6 wt.%), oxygen (43.4 wt.%), phosphorus (14.6 wt.%), and nitrogen (15.2 wt.%). Figure 8b displays the FESEM micrograph, revealing a combination of smooth and flake-like surface features in the ZnMS/APP FRG. This reflects the inherent morphology of the ZnMS while highlighting the surface decoration by APP. The elemental mapping further confirms the homogeneous distribution of N, O, Zn, and P, indicating a correct integration between components. This supports the existence of Zn–O–P coordination bonds, along with physical interactions, both of which contribute to improved thermal stability and char formation of this FRG, which are key features for synergistic flame-retardant performance.

### 3.3. PP-FRG Composites

#### 3.3.1. Chemical Composition (FTIR) and Thermal Behavior (DSC/TGA)

**Figure 9:**
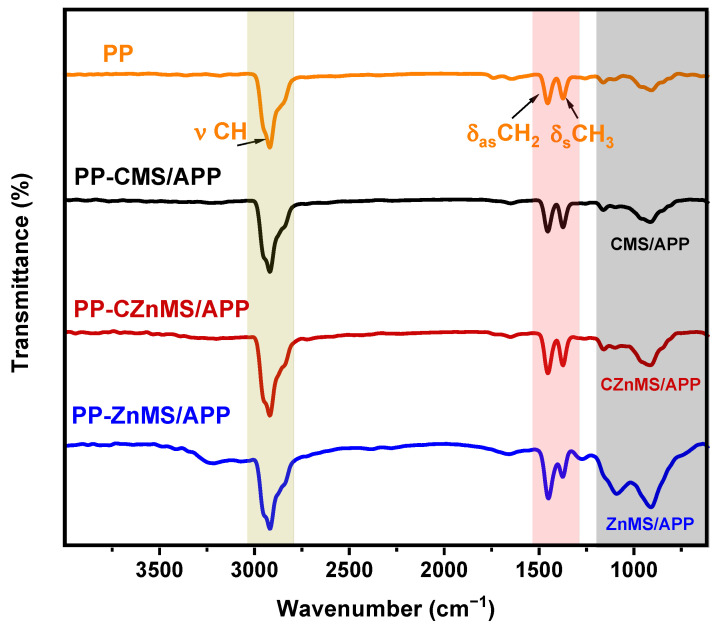
Chemical composition analysis by Fourier transform infrared (FTIR) spectroscopy of neat PP and PP-FRG composites containing CMS/APP, CZnMS/APP, and ZnMS/APP. Gray-shaded regions indicate spectral variations associated with interactions between FRG components and the PP matrix.

The newly developed FRG were incorporated at 10 wt.% into a PP matrix via a melt mixing process to obtain PP-FRG composites. Depending on the FRG type, the formulations were labeled as PP-CMS/APP, PP-CZnMS/APP and PP/ZnMS/APP. The FTIR spectra of neat PP and the composite are shown in Figure 9. The characteristic absorption bands of PP are present not only in the neat polymer but also in all composite samples. The C-H stretching bands appear in the range of 2990–2790 cm^−1^, while asymmetric and symmetric bending vibrations of the CH_2_ and CH_3_ groups are observed at 1457 cm^−1^ and 1370 cm^−1^, respectively [55]. In comparison to the neat PP, the spectra of PP-CMS/APP, PP-CZnMS/APP, and PP-ZnMS/APP do not display prominent new bands above 1200 cm^−1^ that would correspond to distinct flame-retardant components due to the low concentration of FRG. However, within the lower wavenumber region of the spectra, changes in the shape and intensity of the absorption bands are observed. These modifications, highlighted in the gray-shaded area, are likely due to chemical or physical interactions between the microspheres and the amino and phosphate groups of APP, such as hydrogen bonding in the CMS/APP, Zn-O-P coordination in ZnMS/APP, and a combination of both in the CZnMS/APP formulation, such as was discussed in the FTIR spectra (Figure 4) for this flame-retardant grenades. Such spectral variations suggest good compatibility between the FRGs and the PP, allowing the polymer to preserve its intrinsic properties. This compatibility may contribute to improved flame-retardant performance by enhancing the thermal barrier effect and promoting char stabilization.

**Figure 10:**
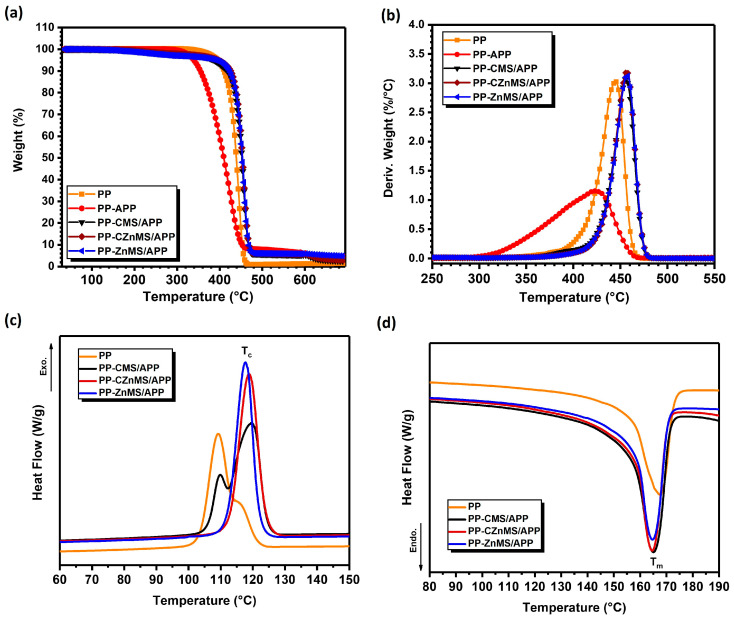
Thermal behavior of neat PP and PP-FRG composites containing CMS/APP, CZnMS/APP, and ZnMS/APP: (**a**) thermogravimetric analysis (TGA) curves, (**b**) derivative thermogravimetric analysis (DTGA) curves; (**c**) crystallization exotherms, and (**d**) melting endotherms obtained by differential scanning calorimetry (DSC).

The thermal properties of the PP-FRG composites were analyzed by TGA (Figure 10a,b), and DSC (Figure 10c,d). The TGA curve for neat PP shows that the polymer remains thermally stable up to approximately 330 °C, after which it undergoes a single-step degradation process, with a maximum weight loss rate observed at 445 °C (as seen in the DTGA curve) and no residual mass at the end of the analysis. For this specific characterization, a composite formulated with only PP and APP was evaluated. In this case, the onset of degradation occurs at a lower temperature (275 °C), and the maximum weight loss rate is reduced by 22 °C, occurring at 423 °C. This behavior is likely due to the poor compatibility between APP and PP, related to their different chemical environments. The premature thermal decomposition of APP appears to accelerate the degradation of the PP backbone. However, this formulation does result in a slightly higher char residue (4.5 wt.%) compared to neat PP, which leaves almost no residue [7,56]. Incorporating FRG; CMS/APP, CZnMS/APP, and ZnMS/APP into the PP matrix seems to induce early degradation too, attributed to the thermal decomposition of the FRG components. Nonetheless, the PP-FRG composites exhibit improved thermal stability in the 350–500 °C range. These composites also yield higher char residues, 2.5 wt.%, 3.0 wt.%, and 5.0 wt.% for PP-CMS/APP, PP-CZnMS/APP, and PP-ZnMS/APP, respectively, compared to the negligible residue (<1 wt.%) of neat PP at 700 °C. DTGA curves highlight the difference in the shift temperature decomposition behavior, displaying peaks at higher temperatures, which imply a more efficient thermal stabilization in the PP-FRG composites indicating that the presence of the FRG in the formulation offers barrier flame retardant efficiency, having maximum weight loss rates at 455 °C, 456 °C and 457 °C for PP-CMS/APP, PP-CZnMS/APP and PP-ZnMS/APP, respectively, which is better than that for neat PP (445 °C) [56].

As part of the thermal behavior evaluation of the PP-FRG composites, differential scanning calorimetry (DSC) was conducted to assess the influence of the incorporated FRG on the thermal transitions of PP. Both the cooling and second heating cycles were analyzed to determine the crystallization temperature (Tc), melting temperature (Tm), and degree of crystallinity (Xc %). The exothermic crystallization curves are shown in Figure 10c, while the endothermic melting curves are presented in Figure 10d. The crystallization profile of neat PP displays a main peak at 109.2 °C and a secondary peak that appears as a shoulder at approximately 116 °C, reflecting heterogeneous crystal populations. Incorporation of the CMS/APP FRG also results in heterogeneous crystallization, but with the main peak shifted to a higher temperature (Tc = 119.5 °C), likely due to a nucleating effect induced by the FRG.

This ability to favor the crystalline domains organization of PP or other thermoplastics, such as PET, during the cooling from the melting step is consistent with previous reports involving carbonaceous materials [14,57]. For the composites with CZnMS/APP FRG and ZnMS/APP FRG, sharp and well-defined crystallization peaks appear at 118.7 °C and 117.7 °C, respectively. These single and sharper peaks suggest the presence of a more homogeneous crystal population, and the increase in the Tc values compared to neat PP confirms the effective nucleation effect of the FRG, reflecting the promotion of earlier and more uniform polymer chain organization during crystallization.

On the other hand, the melting endotherms (Figure 10d) show that all PP-FRG composites exhibit Tm values close to that of neat PP (Tm = 167.5 °C), ranging between 164–165 °C. However, slight variations in peak shape suggest differences in melting enthalpies (ΔHf), and consequently, in the degree of crystallinity (Xc). The ΔHf values were 109.9 J/g for neat PP, 146.8 J/g, 138.6 J/g, and 130.8 J/g for PP-CMS/APP, PP-CZnMS/APP, and PP-ZnMS/APP FRG, respectively. Using a reference ΔHf of 207 J/g for 100% crystalline PP [58], the calculated Xc values were 53.1%, 70.9%, 66.9%, and 63.2%, respectively. The broader Tc and Tm peaks observed in the PP-CMS/APP composite suggest higher crystallinity, but with less perfection or more heterogeneous crystalline domains. In contrast, the composites formulated with CZnMS/APP and ZnMS/APP exhibit higher crystallinity, indicating that the crystal growth was favored, therefore a better crystalline perfection, likely due to more efficient nucleation.

Overall, these thermal behavior results indicate that the presence of the FRG, particularly CZnMS/APP and ZnMS/APP, not only enhances thermal stability and char formation, but also promotes higher crystallinity without significantly altering the intrinsic melting behavior of PP. These features give first support to the hypothesis that combining PP with these newly developed FRG generates a synergistic effect, improving thermal resistance and contributing to enhanced flame-retardant performance.

#### 3.3.2. Morphological Features of PP-FRG Composites

**Figure 11:**
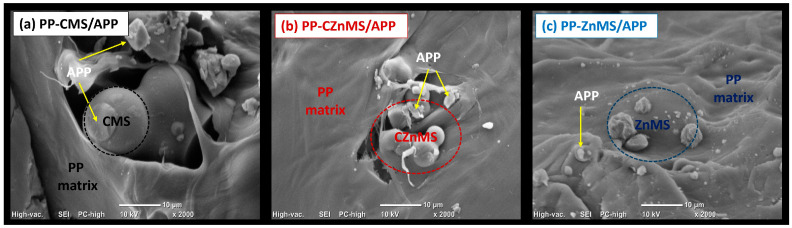
Scanning electron microscopy (SEM) images of the different PP-FRG composites. The specimens were cryo-fractured and coated with Au/Pd prior to analysis. (**a**) PP-CMS/APP; (**b**) PP-CZnMS/APP and (**c**) PP-ZnMS/APP.

To further investigate the dispersion of the newly developed FRG within the polymer matrix, scanning electron microscopy (SEM) was performed on the cryo-fractured surfaces of the composite specimens. In the PP-CMS/APP FRG composite (Figure 11a), the FRG appear embedded within the PP matrix. The CMS retain their spherical morphology, and plate-like APP structures are also visible, consistent with the morphology of the isolated FRG (Figure 6). This microstructure indicates good physical interaction between components, which likely contributes to the nucleating effect observed in the DSC crystallization analysis. The PP-CZnMS/APP composite (Figure 11b) exhibits a uniform distribution of FRG particles, preserving their original morphology. The arrangement appears more compact and cohesive than with CMS/APP FRG, suggesting both physical and chemical interactions that may enhance dispersion and interfacial adhesion. These features are consistent with the improved thermal stability and homogeneous crystallinity detected by TGA and DSC, respectively. Figure 11c presents the SEM image of the PP-ZnMS/APP composite. The FRG, with APP visibly decorating the surface of the ZnMS, appears well distributed throughout the PP matrix. The homogeneous dispersion and absence of interfacial voids indicate strong compatibility with the polymer phase. This morphology correlates with the improvements in thermal resistance (TGA), enhanced crystallinity (DSC), and the possible Zn-O-P bond coordination observed in FTIR analysis.

Overall, SEM analysis supports and complements the FTIR, TGA, and DSC results. The incorporation of FRG, particularly those based on ZnMS/APP and CZnMS/APP, provides structural reinforcement and promotes a synergistic effect, improving thermal stability, crystal development, and flame-retardant potential in PP composites.

#### 3.3.3. Fire Resistance and Mechanical Behavior of PP-FRG Composites

To evaluate the flame-retardant performance of the PP-FRG composites, flammability tests were conducted under the UL-94 horizontal burning standard. The results, along with mechanical properties (flexural strength and modulus), are summarized in Table 2. Neat PP exhibited the highest burning velocity (30.6 mm/min); despite its limited flame-retardant performance, it still qualifies as HB under UL-94, since its burning rate is below the 40 mm/min threshold required for samples thicker than 3 mm. The UL-94 rating for the composites PP-CMS/APP, PP-CZnMS/APP, and PP-ZnMS/APP was also HB; however, all of them showed significantly reduced burning velocities 43–60% compared to neat PP, as well as less dripping of burning molten material, since when the drop is released, it is immediately extinguished, indicating enhanced fire-retardant behavior. The most notable improvement was observed in the PP-CMS/APP composite, which achieved the lowest burning velocity (12.24 mm/min). This is likely due to the synergistic interaction between CMS and APP, which promotes char formation and acts as an effective thermal barrier. This is attributed to the joint capacity of APP to trap combustible gases through decomposition, as well as its capacity to generate char in conjunction with CMS.

The PP-CZnMS/APP and PP-ZnMS/APP composites also demonstrated improved flame retardancy, with burning velocities of 17.32 mm/min and 16.11 mm/min, respectively. These improvements align with the previous TGA results, which showed enhanced char yield and thermal insulation, indicating that the presence of FRG reduces the energy required to sustain the combustion reaction [16,40,43,57]. In both cases, the incorporation of zinc oxide contributes to the formation of a thermally stable protective layer, leading to improved flame-retardant performance. However, the presence of ZnO may also reduce the interaction between APP and the polymer or carbonaceous components, thereby hindering the development of a homogeneous char-resistant layer that functions as an effective fire barrier, as observed in the PP-CMS/APP composite. In the case of PP-ZnMS/APP, the main advantage lies in the increased char residue and reduced dripping of PP and reducing the presence of burning droplets. Still, it can be observed that when a burning droplet is released, the fire is extinguished immediately after it separates from the sample, due to the FRG components inhibiting the fire, although with a lower amount of char. This behavior has the disadvantage of forming a less homogeneous layer compared to PP-CMS/APP.

Regarding mechanical performance, all PP-FRG composites preserve or slightly enhance the flexural strength relative to neat PP, indicating that the addition of the FRG does not compromise polymer structural integrity or its processing. More significantly, notable increases in flexural modulus were observed: 37.5% for PP-CMS/APP, 12.6% for PP-CZnMS/APP, and 9.3% for PP-ZnMS/APP. These improvements suggest that the FRG acts as a reinforcing filler, contributing to stiffness by facilitating stress transfer and enhancing interfacial adhesion [55,59,60]. These findings correlate with the adequate dispersion and good physical interaction observed in SEM micrographs between the PP matrix and the FRG. In contrast, when flame retardants agglomerate within the matrix, as reported by Hu et al. [20], for PP with xylose-derived CMS decorated with layered double hydroxide, mechanical performance can deteriorate. Therefore, in our case, both the mechanical properties and UL-94 flammability results support the previous discussion, indicating that the incorporation of the newly developed FRG contributes to a synergistic enhancement in both fire resistance and mechanical performance.

**Figure 12:**
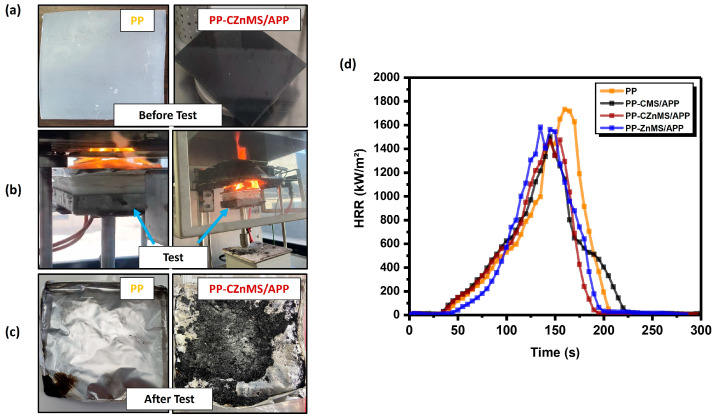
Cone calorimetric test (CCT) results for neat PP and PP-flame-retardant grenade (PP-FRG) composites: (**a**) Optical images of PP and PP-CZnMS/APP specimens before testing, (**b**) real-time combustion testing in progress, and (**c**) post-test chart residue; (**d**) heat release rate (HRR) curves for all evaluated composites.

Following the ASTM E1354 standard, cone calorimetric tests (CCTs) were conducted to simulate the combustion behavior of neat PP and the composites containing the different flame-retardant grenades, CMS/APP, CZnMS/APP and ZnMS/APP, under real-scale fire conditions. Figure 12a displays representative optical images of square plate specimens of neat PP and the PP-CZnMS/APP composite before combustion, during real-time testing, and after the test. These images reveal that while neat PP undergoes complete degradation, the PP-CZnMS/APP composite forms a substantial char layer, suggesting the establishment of a thermal barrier effect. Figure 12b shows the HRR curves generated during testing. At the same time, key combustion parameters such as heat release rate peak (PHRR), total heat release (THR), time to ignition (TTI), and char residue percentage are summarized in Table 3.

Neat PP exhibited the highest PHRR and THR values at 1734.1 kW/m^2^ and 118.3 MJ/m^2^, respectively, with a TTI of 35 s and leaving negligible chart residue. These poor fire-resistance characteristics are typical of unmodified PP and are attributed to their fully hydrocarbon backbone, lacking aromatic structures or heteroatoms that could promote char formation or thermal stability [3]. The incorporation of the synthesized FRG significantly improved flame-retardant performance, as evidenced by the reduction in both PHRR and THR. Specifically, the PHRR was reduced by approximately 13%, 15%, and 9% for CMS/APP, CZnMS/APP, and ZnMS/APP, respectively, indicating that the FRG formed an insulating barrier that limited heat and oxygen transfer. THR values also decreased across all composites, with PP-CZnMS/APP exhibiting the lowest value (100.7 MJ/m^2^), suggesting reduced combustion and enhanced barrier formation due to the higher chart yield promoted by the synergistic action of carbon, APP and Zinc. These improvements align with the UL-94 test results and mechanical property enhancements, reinforcing the importance of proper dispersion and interaction of FRG acting as fillers within the polymer matrix in the PP-FRG composites. Similar behavior has been reported in EVA-graphite and LDPE-graphite systems, where well-dispersed fillers lead to the formation of a continuous protective layer that insulates the polymer from heat, retarding the combustion [60].

On the other hand, although TTI values did not show a clear trend (ranging from 30 to 38 s), the PP-CZnMS/APP and PP-CMS/APP composites, despite slightly earlier ignition, demonstrated superior char formation and thermal stability. This suggests that early ignition does not necessarily compromise flame-retardant performance if effective barrier mechanisms are activated post-ignition. Therefore, considering all CCT parameters, the best-balanced performance as a flame retardant with a synergistic effect was accomplished when CZnMS/APP was incorporated in the PP-FRG composites. The presence of CZnMS decorated with APP appears to enhance interaction with the PP matrix, potentially promoting slight intumescence and forming a more effective thermal barrier, reducing the dripping of burning polymer. These findings are consistent with TGA evidence of greater char yields and higher thermal stability, confirming the potential of these novel FRGs as multifunctional additives for fire-safe thermoplastics, such as PP.

## 4. Conclusions

Various microspheres, including CMSs, CZnMSs, and ZnMSs, were successfully synthesized via a hydrothermal method, which is sustainable, versatile, and cost-effective. Their formation was confirmed by FTIR and UV-Vis, which identified characteristic absorption bands for each system. XRD revealed their respective amorphous (CMS), partially semicrystalline (CZnMS), and crystalline structures (ZnMS). TGA analysis confirmed their thermal stability and ability to generate char, while SEM observations evidenced their spherical morphology with size variations depending on composition. These microspheres were then combined with APP to create novel synergistic flame-retardant systems, referred to as FRG. Comprehensive characterization (FTIR, TGA, FESEM, EDX) revealed a progressive interaction mechanism: from physical adsorption in CMS/APP to combined physical and covalent bonding in CZnMS/APP and strong chemical coordination in ZnMS/APP. The FRGs were incorporated into PP via melt mixing to obtain PP-CMS/APP, PP-CZnMS/APP, and PP-ZnMS/APP composites. FTIR spectra and SEM micrographs confirmed good compatibility between components, while TGA showed increased thermal stability, raising the temperature of the maximum mass loss rate by over 10 °C compared to neat PP, along with an increased char yield. DSC analysis indicated earlier, and more uniform crystallization (higher Tc values) of PP. UL-94 tests confirmed HB classification, with burning rates reduced by up to 43–60% using only 10% of GRF. This demonstrates the potential of using these GRFs at higher concentrations to mitigate fire in PP, as evidenced by the cone calorimetry test, which showed reductions in PHRR and THR of 1734.1 to 1475.4 kW/m^2^ and 118.3 to 100.7 MJ/m^2^, respectively. Mechanical testing revealed improved flexural modulus and preserved or slightly increased flexural strength. Overall, the incorporation of these novel FRGs imparted a synergistic flame-retardant effect, yielding PP composites with enhanced fire resistance and mechanical integrity at a low FRG concentration (10 wt.%), providing a viable route for the development of fire-safe PP materials.

## Figures and Tables

**Table 1:** Formulation of different PP-FRG composites.

Sample	Polymer	FR *	FRG *	
	PP * (wt.%)	APP * (wt.%)	C * (wt.%)	Zn * (wt.%)
**PP**	100	**-**	**-**	**-**
**PP-APP**	90	10	**-**	**-**
**PP-CMS/APP**	90	8.3	1.7	**-**
**PP-CZnMS/APP**	90	8.3	0.85	0.85
**PP-ZnMS/APP**	90	8.3	**-**	1.7

* PP = Polypropylene; APP = ammonium polyphosphate; FR = Flame retardant; FRG = Flame-Retardant Grenades; C = carbon source microspheres; Zn = zinc oxide source microspheres.

**Table 2:** UL-94 flammability test results and flexural mechanical properties of neat PP and PP-FRG composites.

Sample	Burning Velocity	Classification	Flexural Strength	* Flexural Modulus
	mm/min	UL-94	MPa	MPa
**PP**	30.6	HB	39.6 ± 2	1076.3
**PP-CMS/APP**	12.24	HB	47.1 ± 3	1480.3
**PP-CZnMS/APP**	17.32	HB	40.1 ± 2	1212
**PP-ZnMS/APP**	16.11	HB	43.3 ± 2	1176.2

* Determined within the linear region at strains below 5%. Average standard deviation for flexural modulus ±65 MPa.

**Table 3:** Cone calorimetric test results for neat PP and PP-FRG composites.

Sample	PEAK HRR	THR	TTI	Residue
	kW/m^2^	MJ/m^2^	s	%
**PP**	1734.1	118.3	35	0
**PP-CMS/APP**	1501.9	112.0	30	7
**PP-CZnMS/APP**	1475.4	100.7	31	7.1
**PP-ZnMS/APP**	1582.7	105.7	38	6.9

## Data Availability

The data that support the findings of this study are presented in this article/Supplementary Material. Further inquiries can be directed to the corresponding authors.

## Supplementary Materials

The following supporting information can be downloaded at: https://www.mdpi.com/article/10.3390/polym17212878/s1, Figure S1: Schematic illustration and optical micrographs of the particle size reduction process of ammonium polyphosphate (APP) using ultrasonic treatment. The left panels show the as-received APP particles and their size distribution; the right panels show APP particles after ultrasonic treatment, filtration, and lyophilization, exhibiting reduced average particle size.
