# Supplementary material for: Synthesis of Carbon-Zinc Oxide Microspheres Decorated with Ammonium Polyphosphate (APP) for Synergistic Flame Retardancy in Polypropylene Composites
Source: Polymers (Basel). 2025 Oct 29;17(21):2878. doi: 10.3390/polym17212878 (PMC12609974; doi:10.3390/polym17212878)
Supplement: Supplementary file 1 [file polymers-17-02878-s001.zip › polymers-3896055-supplementary.pdf]

*Supplementary information*

*Article*

# Synthesis of Carbon-Zinc Oxide Microspheres Decorated with Ammonium Polyphosphate (APP) for Synergistic Flame Retardancy in Polypropylene Composites

**Juan J. Mendoza [1,2] , Jesús R. Campos [1] , Ramón Enrique Díaz de León - Gómez [1] , Luciano da Silva [1] , Antonio Serguei Ledezma-Pérez [1] , Arxel de León [3]* Edgar Nazareo Cabrera-Álvarez [4]***

[1] Centro de Investigación en Química Aplicada (CIQA), Blvd. Enrique Reyna #140, Col. San José de Los Cerritos, C.P. 25294, Saltillo, Coahuila, México.

[2] Secretaría de Ciencia, Humanidades, Tecnología e Innovación (SECIHTI), Av. Insurgentes Sur 1582, Col. Crédito. Constructor, Demarcación Territorial Benito Juárez. CP 03940, Ciudad de México.

[3] SECIHTI-CIQA, Blvd. Enrique Reyna #140, Col. San José de Los Cerritos, C.P. 25294, Saltillo, Coahuila, México.

[4] SECIHTI-CIQA Monterrey, Alianza Sur 204, PIIT Nuevo León C.P., Apodaca, 66628, México.

* Correspondence: edgar.cabrera@ciqa.edu.mx (E.N.C.A.); arxel.deleon@ciqa.edu.mx (A.de L.); Tel. +52 8444389830-1226 (E.N.C.A.)

### Size reduction of ammonium polyphosphate (APP) particles

A 2 wt.% suspension of commercial APP was prepared by dispersing the powder in 100 mL of deionized water under continuous stirring at 500 rpm. The suspension was then subjected to ultrasonication using a probe at 33.7 μm amplitude (60% power) for 30 min to reduce the particle size. The treated suspension was filtered through an 11 μm pore size membrane to remove larger particles and subsequently dried via lyophilization.

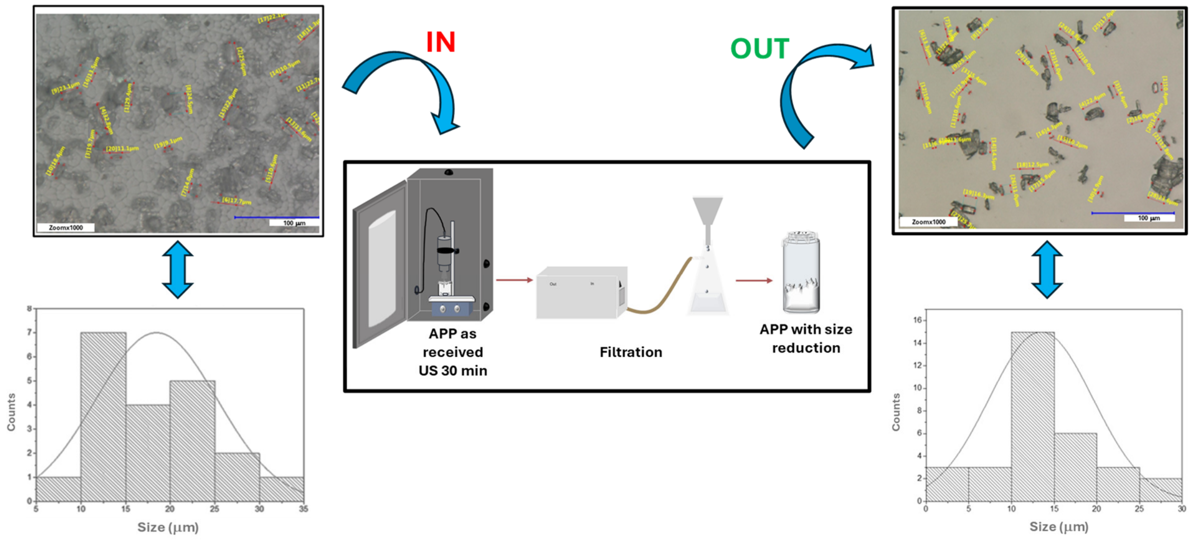


**Figure S1.** Schematic illustration and optical micrographs of the particle size reduction process of ammonium polyphosphate (APP) using ultrasonic treatment. The left panels show the as-received APP particles and their size distribution; the right panels show APP particles after ultrasonic treatment, filtration, and lyophilization, exhibiting reduced average particle size.
